# The role of rotational hand movements and general motor ability in children’s mental rotation performance

**DOI:** 10.3389/fpsyg.2015.00984

**Published:** 2015-07-16

**Authors:** Petra Jansen, Jan Kellner

**Affiliations:** Institute of Sport Science, University of Regensburg, Regensburg, Germany

**Keywords:** children, mental rotation, motor processes, motor ability, interference

## Abstract

Mental rotation of visual images of body parts and abstract shapes can be influenced by simultaneous motor activity. Children in particular have a strong coupling between motor and cognitive processes. We investigated the influence of a rotational hand movement performed by rotating a knob on mental rotation performance in primary school-age children (*N* = 83; age range: 7.0–8.3 and 9.0–10.11 years). In addition, we assessed the role of motor ability in this relationship. Boys in the 7- to 8-year-old group were faster when mentally and manually rotating in the same direction than in the opposite direction. For girls and older children this effect was not found. A positive relationship was found between motor ability and accuracy on the mental rotation task: stronger motor ability related to improved mental rotation performance. In both age groups, children with more advanced motor abilities were more likely to adopt motor processes to solve mental rotation tasks if the mental rotation task was primed by a motor task. Our evidence supports the idea that an overlap between motor and visual cognitive processes in children is influenced by motor ability.

## Introduction

The focus of this study is the investigation of motor processes, motor ability, and mental rotation in primary school-age children. Mental rotation is the ability to imagine how a stimulus would look when rotated (Shepard and Metzler, 1971). Motor processes may be investigated by analyzing how participants conduct particular movements (e.g., rotating a handle). Motor ability is evaluated based on participants’ level of performance on particular motor tasks (e.g., coordination).

### Mental Rotation in Adults and Children

The original paradigm to test mental rotation ability was developed by Shepard and Metzler (1971). In this paradigm, participants have to discriminate as fast and accurately as possible whether a rotated figure is identical or a mirror reversed image of an original upright figure. Response times (RTs) in this paradigm typically show a linear increase with increasing angular disparity, which indicates that participants mentally rotate one figure into congruence with the upright position of the other figure before making a decision (Courbois, 2000). It has been concluded that mental transformations are subject to the same spatio-temporal constraints as perceived movements in the external world (Metzler and Shepard, 1982). A frequent phenomenon observed in mental rotation is a gender difference favoring males (Voyer et al., 1995). This effect can also be found in primary school-age children (Jansen et al., 2013). There is good evidence for psychosocial (Moè and Pazzaglia, 2006; Nazareth et al., 2013) as well as biological-neuronal (McGlone, 1980; Imperato-McGinley et al., 1991) explanations for this difference. A complex interaction of these factors seems to be responsible for males outperforming females on mental rotation tasks.

Research concerning the development of mental rotation ability in children has shown that at 4 years of age, some children are already able to mentally rotate age-appropriate stimuli, such as pictures of toy bears (Marmor, 1975; Estes, 1998). By the age of five (Kosslyn et al., 1990) or six (Estes, 1998), most children can mentally rotate more complex figures, especially after receiving training (for an overview, see Newcombe and Frick, 2010; Frick et al., 2014). However, mental rotation in children as young as five seems to depend on the characteristics of the stimuli. Courbois (2000) showed that it was difficult for 5-year-old children to mentally rotate stimuli without salient axes. Generally, mental rotation speed and accuracy (hit rate, HR) increase with age and reach adult levels during adolescence (Kail et al., 1980).

### Motor Processes, Motor Ability, and Mental Rotation in Adults

According to the embodiment approach in cognitive science, simple sensory motor interaction with the environment plays an important role in the development of advanced cognitive skills (Wheeler and Clark, 2008). The viewpoint of embodiment states that cognitive processes are deeply rooted in the body’s interaction with the world and that sensory and motor resources are used for off-line cognitive activity. For example, mentally simulated external events can be used in mental imagery (Wilson, 2002) and gestures can help mental rotation performance (Chu and Kita, 2011). A vast body of literature has investigated the relationship between physical activity, motor skills, and cognitive skills. Mental rotation is one prominent paradigm used to explore the link between body and mind. This is because mental rotation—which requires all basic spatial abilities (Linn and Petersen, 1985)—makes comprehensive demands on mental abilities. If there is a link between body and mind, it should be rather evident in more difficult tasks than in simpler tasks, which do not exploit mental capacity. According to Kosslyn et al. (2001) there are at least two distinct mechanisms used to rotate objects, one that involves motor processing and one that does not. To further support this idea, it has been shown that the use of motor processes can be implicitly manipulated via the introduction of motor content prior to or during mental rotation (Wraga et al., 2003).

The relationship between motor and mental rotation processes has been investigated using different approaches. One approach explores the effect of physical activity on mental rotation ability. For example, Moreau et al. (2012) investigated the effect of 10 months of wrestling training compared to 10 months of running training. They found that wrestlers showed a significant improvement in mental rotation performance compared to runners.

A second approach investigates the motor processes used while solving a mental rotation task. In several studies with adults it has been shown that anatomical restraints affect the mental rotation of visual images of body parts (Sekiyama, 1982; Parsons, 1987; Pellizzer and Georgopoulos, 1993) and other stimuli (e.g., abstract shapes; Chu and Kita, 2011). Chu and Kita (2011) found better mental rotation performance when participants were encouraged to use supportive motor gestures while solving a mental rotation task with cube figures as stimuli compared to participants who were told to sit on their hands. The advantage in mental rotation for the gesture group continued even if the use of gestures was prevented in a subsequent block. The authors ascribe the effect to an internalization of the gestures and propose that gesture improves the internal computation of spatial transformation in a general way. In the quasi-experimental study by Moreau (2012), wrestlers were found to demonstrate better mental rotation performance than runners. However, this advantage disappeared when participants’ hands were restrained. These findings suggest that the wrestler’s advantage in mental rotation of abstract objects is not based on mental rotation ability *per se*, but on the underlying processes for this task, such as action simulation. Thus, the fact that restraining the hands cleared the advantage of the wrestler shows that they used some covert action of the hands to improve mental rotation. Otherwise stated, it is inferred from the degradation of performance that some action simulation (i.e., covert hand movement) must have taken place in the condition without the hands restrained to improve performance compared to non-wrestlers.

A third approach is to look at the relationship between a motor task and a mental rotation task by using an interference paradigm, in which a motor and a mental rotation must be conducted simultaneously. Concurrent motor rotation included rotating a knob while mentally rotating a stimulus in the same or the opposite direction, which should evoke the involvement of motor processes in mental rotation (Wraga et al., 2003; Chu and Kita, 2011). Using this technique, Wohlschläger and Wohlschläger (1998) found that motor and mental rotation share common processes: Congruent manual and mental rotation improved mental rotation performance, whereas incongruent manual and mental rotations (i.e., rotations in opposite directions) degraded mental rotation performance. A similar result was shown in the interference study of Wexler et al. (1998). Wohlschläger (2001) demonstrated this interference effect even when participants only had the intention of manually rotating a knob (but without a real motor task) while performing a mental rotation task.

Considering these three approaches, mental rotation of images of bodies or body parts, and even abstract objects, automatically engage embodiment processes (Krüger et al., 2014) and might be supported or disturbed by the use of covert motor processes. Experts in motor rotation rely more automatically on covert motor rotations when mentally rotating abstract stimuli (Moreau, 2013). For children, this relationship between motor processes, motor abilities, and mental rotation has yet to be investigated thoroughly, but some important work has been conducted.

### Motor Processes, Motor Ability, and Mental Rotation in Children

Jansen and Heil (2010) found a relationship between motor ability and mental rotation skills in 5- to 6-year-old children. Motor abilities including a coordinative component (e.g., collecting matches or sticks bimanually) were a strong predictor for mental rotation performance. Ehrlich et al. (2006) confirmed the relation between gestures and spatial transformation tasks for children as young as 5 years. In comparison to adults, the connection between motor processes and the rotation of mentally represented objects seems to be stronger in children. Frick et al. (2009) showed an interference effect between motor rotation and a simultaneous mental rotation task for children less than 9 years of age. The study included four age groups: 5-year-olds, 8-year-olds, 11-year-olds, and adults. Figure and ground pairs were used as stimuli to avoid ambiguity of the direction of mental rotation. The motor rotation was carried out by turning a wheel with a handle. In older children (11-year-olds) and adults, interference was not detected. Based on these results it was concluded that the ability to differentiate between motor processes investigated by a concurrent motor task and cognitive processes develops with age. In another study, Funk et al. (2005) found a stronger involvement of motor processes for the mental rotation of images of hands in 5- to 7-year-old children than in adults. Krüger and Krist (2009) also found an effect of motor processes in the mental rotation of images of hands to be stronger in first graders than in adults.

### Goal and Hypotheses of the Present Study

The main goal of this study was to investigate whether motor and mental rotation share common processes according to the studies of adults by Wohlschläger and Wohlschläger (1998) and of children by Frick et al. (2009). In addition, we aimed to investigate whether those common processes depend on the motor ability of primary school-age children (Jansen and Heil, 2010). In doing so, we integrate two different approaches for the study of motor effects on mental rotation for the first time in this age group.

Our paradigm was similar to that used by Frick et al. (2009) but with some important differences. Instead of using figure ground pairs as stimuli for the mental rotation task, we used a classic mental rotation paradigm with two stimuli presented side by side. Because cube figures have been shown to be too difficult for 7- to 8-year-old children (Jansen et al., 2013), we used animal figures which were rotated in the picture plane. Rotation in the picture plane was chosen to ensure that the manual and mental rotation used the same axis. For manual rotation, a rotating knob of approximately the same size as the depicted animal figures was used. We tried to match the assumed covert motor process and the real motor process as closely as possible. In addition, we chose to use more trials in comparison to Frick et al. (2009) in each condition and to test more participants in each age group to increase the reliability of our data and to be able to draw conclusions about a possible gender effect.

We expected to find 9- to 10-year-old children to be superior to 7- to 8-year-old children in mental rotation performance. We expected to find interference effects between manual and mental rotation in the younger age group manifested by longer RTs and lower accuracy (HR) for incompatible versus compatible manual and mental rotation.

Since mental rotation performance is often related to motor abilities (Jansen and Heil, 2010; Jansen et al., 2011b), each child completed a motor test, measuring manual dexterity, balance and ball skills. According to the study of Moreau (2012) with adults, we hypothesized that children with stronger motor skills would rely more on the beneficial involvement of motor processes while solving mental rotation tasks. Therefore, we expected to find a positive relationship between motor abilities and mental rotation performance. In addition, we anticipated an interaction between motor ability and the compatibility of manual and mental rotation. Manual and mental rotations are compatible when animal picture and knob are rotated in the same direction. If children with increased motor ability rely more on motor processes when mentally rotating, a simultaneously executed incompatible motor rotation should be more distracting for these children than for those with poorer motor ability. Additionally, we anticipated a priming effect that would result in a stronger correlation between mental rotation performance and motor ability in the experimental block that followed trials on which mental and manual rotation were combined. Finally, we expected to find an interaction between this type of motor priming and children’s motor ability.

Although gender differences were not the main focus of the study, we predicted, according to Jansen et al. (2013), a gender difference in mental rotation performance with boys outperforming girls. We did not know, however, how gender related to the possible motor interference effect.

## Materials and Methods

### Participants

In this study, 83 children in two age groups were tested at their schools: 45 children were in the 7- to 8-year-old age group (range: 7.0–8.3 years; *M* = 7.7; SD = 0.3; male: 21, female: 24) and 38 children were in the 9- to 10-year-old age group (range: 9.0–10.11 years; *M* = 9.8; SD = 0.5; male: 18, female: 20). Children were recruited from two primary schools. All parents were informed that the experiment was conducted in accordance with the Ethical standards of the APA and gave written informed consent. Participants had normal or corrected-to-normal vision and 77 were right-handed. Six children (5.3%) were left-handed, however, due to this low percentage neither a separate analysis nor a modified experiment was conducted for the left-handed group.

### Apparatus and Stimuli

All children completed the Movement Assessment Battery 2 for children (M-ABC-2; Petermann, 2008) and a chronometric mental rotation test with and without concurrent manual rotation.

#### Movement Assessment Battery

The M-ABC-2 (Petermann, 2008) assesses sensory-motor ability in three dimensions: hand dexterity, ball skills, and balance. The test was chosen because it covers relevant motor areas, which correlate with mental rotation performance in children (Jansen and Heil, 2010; Jansen et al., 2011a). Two weeks test-retest reliability for this test is given with *r* = 0.97 in the handbook. The inter-rater-reliability specified is 0.95. Thus, the M-ABC-2 is a reliable means to assess motor ability in children.

The hand dexterity assessment included three tests: placing pegs in a board with holes, threading a lace through a lacing board, and drawing a trail. The ball skills assessment included catching a ball bounced off a wall with two hands and throwing a bean bag onto a mat 1.8 m away. The balance assessment consisted of one-legged balancing on a balance board, walking heel-to-toe forward, and one-legged hopping on mats.

An overall score was used for statistical analysis. Children reached an overall composite score of *M* = 10.94 (SD = 2.72), which equals a percentile rank of 60 (generally, composite scores can range from 1 to 19). There were no significant differences between age groups or sexes (all *p* > 0.05).

#### Chronometric Mental Rotation Test with Additional Manual Rotation

***Chronometric mental rotation test***

Testing was carried out on laptop computers (15-inch monitor) with a rotating knob in a box connected to the laptop. Children were seated at a table with the laptop in front of them. Stimuli for the mental rotation test were presented using the software Presentation (Neurobehavioral Systems). The stimuli consisted of nine different animal pictures (Snodgrass and Vanderwart, 1980): alligator, bear, cat, dog, donkey, elephant, fox, gorilla, and rabbit. Each picture was 7 cm × 7 cm on the screen and the two images were spaced 5 cm apart. Participants were free to choose the most comfortable viewing distance. Two stimuli were presented on the screen simultaneously. The right stimulus was either identical to the left or mirror-reversed. The left stimulus appeared always upright while the right stimulus was rotated 0°, +45°, +90°, +135°, 180°, –135°, –90°, or –45°. Children were explicitly instructed to mentally rotate the right stimulus to align it with the left, upright stimulus (shown in its canonical orientation). A positive angle corresponded to stimuli rotated in a clockwise direction and a negative angle corresponded to stimuli rotated in a counterclockwise direction.

Children were asked to decide if the two animals on the screen were the same or mirror reversed by way of pressing one of two marked keys (colored red and green) on the keyboard of the laptop. The buttons were the left and right mouse button underneath the touchpad and had to be operated with the forefinger and the middle finger of the left hand. Children had to use the left hand for the button presses in all blocks to avoid differences between conditions with and without concurrent manual rotation. Instructions were given in child appropriate language, i.e., they were told to mentally rotate the right animal the shortest way (regarding rotation angle) until it was standing on its feet like the left animal and to press the green button if the animals looked in the same direction or the red button if the animals looked in opposing directions. In addition, they were told to respond as quickly and accurately as possible. Only one stimulus pair was used for the practice trials to familiarize children with the demands of the task and eight different stimulus pairs were used for the test trials, resulting in a total of 128 different stimulus pairs: 8 (animals) × 2 (same/mirror reversed) × 8 (angular disparity). The angles in the practice and in the test trials were the same. The two stimuli stayed on the screen until a response was made. The setup was the same for all children, regardless of dominant hand. Following the response a smiling face or frowning face appeared for 1000 ms as feedback. Feedback was used throughout the experiment to maintain motivation.

RT and HRs were analyzed. Trials with RT below 300 ms and over 15000 ms were considered outliers and treated as errors (0.7% of all trials). RTs faster than 300 ms in a mental rotation task are not possible without guessing (Schmidt and Lee, 2011) and the upper limit of 15000 ms was chosen to provide children with more time to make a decision on the demanding interference task. For the RT analysis, only correct responses to non-mirror reversed stimuli were used because angular disparity is not clearly defined for mirror-reversed responses (Jolicøeur et al., 1985). Thus, 128 trials per participant were used in the RT analysis.

***Motor rotation***

The box with the rotating knob was positioned on the table at the right side of the laptop. The knob was 4 cm in diameter and could only be rotated around the z-axis. The dimensions of the box were 14 cm × 15 cm × 35 cm (height × width × length) and the knob was placed inside to prevent participants from seeing their hand turning the knob. The knob approximately matched the size of the animal pictures presented in the mental rotation task. We chose a knob because the rotation resembles the movement of actually picking up an animal figure and turning it.

**FIGURE 1:**
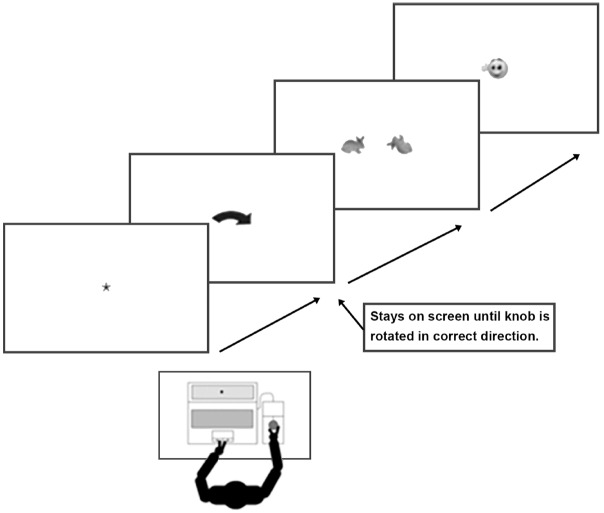
**Schematic drawing of the sequence of stimuli presented within one trial**.

Children turned the knob with their right hand in the manual rotation trials. The fixation cross was followed by a curved arrow indicating the direction the knob should be rotated in. The experiment only proceeded if children turned the knob in the correct direction. The arrow stayed on the screen until the knob was rotated in the correct direction. The mental rotation stimuli appeared as soon as the arrow disappeared and stayed on screen until a response was made (see Figure 1). Children were told to continue rotating the knob until the feedback was shown. The direction of the curved arrow stayed the same for each participant but was randomized in each age group resulting in 22 children rotating the knob clockwise and 23 children rotating counterclockwise for the 7- to 8-year-old group and 19 children rotating the knob clockwise and 19 rotating it counterclockwise for the 9- to 10-year-old group.

### Procedure

The order of the mental rotation test and the M-ABC-2 was counterbalanced. The mental rotation test began with 16 practice trials. The experimental phase consisted of four blocks of 64 trials each. In each block, four different animal pictures were used. The first and fourth blocks consisted of mental rotation only and the second and third blocks consisted of mental and manual rotation. This design was chosen to equally distribute possible training effects.

## Results

In this section, we first describe the results for mental rotation performance. Next, we describe analyses of interference effects between simultaneous manual and mental rotation on RTs and HRs in the mental rotation task. Finally, we investigate if manually rotating a knob in context with mental rotation (as in the second and third block) sheds a light on the relationship between motor ability and mental rotation performance.

### Analysis of Mental Rotation Performance

To investigate children’s performance in mental rotation, RT and HRs were analyzed in all four blocks of the test.

#### Response Time

RT was submitted to an ANOVA with the within-subject factors “angular disparity” (0°, +45°, +90°, +135°, 180°, –135°, –90°, –45°) and “manual rotation” (with and without) and the between-subject factors “age group” (7–8 vs. 9–10), “gender” (male vs. female), and “direction of manual rotation” (clockwise vs. counterclockwise). Main effects were found for “angular disparity,” *F*(7,504) = 82.87, *p* < 0.001, ${\text{η}}_{p}^{2}$ = 0.54, as well as for the factor “age group,” *F*(1,72) = 20.1, *p* < 0.001, ${\text{η}}_{p}^{2}$ = 0.22.

A repeated contrast analysis was run for the factor angular disparity to take a closer look at the differences between each consecutive angle. All contrasts were statistically significant (*p* < 0.05). The respective means (averaged across clockwise and counterclockwise rotation) were 1724.53, 2037.26, 2350.02, 2766.59, and 3039.21 ms for the angles 0°, 45°, 90°, 135°, and 180°. A linear regression analysis was computed to model the relation between rotation angles and RT, which yielded a significant result [*F*(1,4) = 872.63, *p* < 0.001].

The younger children had longer RT than older children (*M* = 2704 ms, SE = 97 vs. *M* = 2063 ms, SE = 105). In addition, a main effect was found for “manual rotation,” *F*(1,72) = 27.35, *p* < 0.001, ${\text{η}}_{p}^{2}$ = 0.28. RT was longer when mental rotation and manual rotation were performed simultaneously (*M* = 2530 ms, SE = 85 vs. *M* = 2238 ms, SE = 67). An interaction also occurred between “angular disparity” and “gender,” *F*(7,504) = 2.6, *p* < 0.05, ${\text{η}}_{p}^{2}$ = 0.04. *Post hoc* analyses with *t*-tests for each angle did not produce any significant differences between boys and girls.

***0° -trials***

To control for effects other than mental rotation, such as perception, encoding of stimuli and motor reaction, an ANOVA for the dependent variable RT in 0° -trials was performed. The within-subject factor was “manual rotation” (with or without) and the between-subject factors were “gender” (male vs. female), “age group” (7–8 vs. 9–10), and “direction of manual rotation” (clockwise vs. counterclockwise). Main effects were found for “age group,” *F*(1,75) = 13, *p* < 0.01, ${\text{η}}_{p}^{2}$ = 0.15, and “manual rotation,” *F*(1,75) = 10.5, *p* < 0.01, ${\text{η}}_{p}^{2}$ = 0.12. Younger children had longer RT than older children (*M* = 1943 ms, SE = 82 vs. *M* = 1509 ms, SE = 89) and RT was shorter when no additional manual rotation had to be performed (*M* = 1602 ms, SE = 51 vs. *M* = 1849 ms, SE = 87). These data suggest that perceptual and motor processes are faster for the older age group in comparison to the younger and are also faster when children perform a spatial task in comparison to a dual task.

***Mental rotation speed***

Mental rotation speed is calculated as the inverted slope of the regression. Its analysis sheds light on the process of mental rotation without the time needed for processes such as perception, encoding of stimuli and motor reaction. Due to negative rotation speed or values more than three standard deviations above or below the mean, four children had to be excluded from the analysis. Afterward mental rotation speed was submitted to an ANOVA with the within-subject factor “manual rotation” and the between-subject factors “age group” and “gender.” A main effect for the factor “gender” was found, *F*(1,71) = 5.25, *p* < 0.05, ${\text{η}}_{p}^{2}$ = 0.07. Boys rotated faster than girls across all age groups (*M* = 192° /s, SE = 13 vs. *M* = 153° /s, SE = 11). No other effects or interactions were found.

#### Hit Rates

HR was submitted to an ANOVA with the within-subject factors “angular disparity” (0°, +45°, +90°, +135°, 180°, –135°, –90°, –45°) and “manual rotation” (with and without) and the between-subject factors “age group” (7–8 vs. 9–10), “gender” (male vs. female) and “direction of manual rotation” (clockwise vs. counterclockwise). Main effects were found for “angular disparity,” *F*(7,525) = 19.72, *p* < 0.001, ${\text{η}}_{p}^{2}$ = 0.21, as well as for the factor “age group,” *F*(1,75) = 5.76, *p* < 0.05, ${\text{η}}_{p}^{2}$ = 0.07. HR decreased with increasing angle (repeated contrast analyses showed that contrasts between 0° and 45°, 135° and 180°, –90° and –45° are significant with *p* > 0.05; all other contrasts *p* < 0.05) and younger children made more errors than older children (*M* = 89%, SE = 1.4 vs. *M* = 94.1%, SE = 1.6). In addition, a main effect was found for “manual rotation,” *F*(1,75) = 7.154, *p* < 0.01, ${\text{η}}_{p}^{2}$ = 0.9. HR was higher when mental rotation and manual rotation were performed simultaneously (*M* = 92.4%, SE = 1 vs. *M* = 90.6, SE = 1.2). An interaction appeared between “manual rotation” and “gender,” *F*(1,75) = 4.82, *p* < 0.05, ${\text{η}}_{p}^{2}$ = 0.06. *Post hoc* analyses with *t*-tests for each condition did not produce any significant differences so this interaction was not analyzed in further detail. Finally, to rule out a possible speed-accuracy tradeoff a correlation analysis between mean HR and mean RT was performed. Only significant negative correlations could be found: mental rotation only (*r* = –0.23, *p* < 0.05), mental and manual rotation (*r* = –0.3, *p* < 0.01), indicating that children with higher HR also had shorter RT.

### Analysis of the Effect of Compatible and Incompatible Manual and Mental Rotation

To investigate whether manual and mental rotation share common underlying processes, the effect of compatible and incompatible manual and mental rotation on RT and HR in the two blocks with manual rotation (block 2 and 3) was investigated. Negative and positive angles were classified as compatible or incompatible according to the participant’s direction of manual rotation. RT and HR for the angles 0° and 180° were excluded from this analysis because either no rotation was needed to solve the task or the direction of rotation was arbitrary. The remaining 48 trails per participant were used in this analysis after excluding the trials with 0° and 180° rotation angle. A 3 (angular disparity) × 2 (compatibility) × 2 (age group) × 2 (gender) × 2 (direction of manual rotation) ANOVA with the dependent variables RT and HR was used. M-ABC-2 score was considered as a covariate in the analysis of HR because partial correlation analyses between M-ABC-2 score, RT and HR in block 2 and 3 only showed significant results for HR (*r* = 0.29, *p* < 0.01).

#### Response Time

**FIGURE 2:**
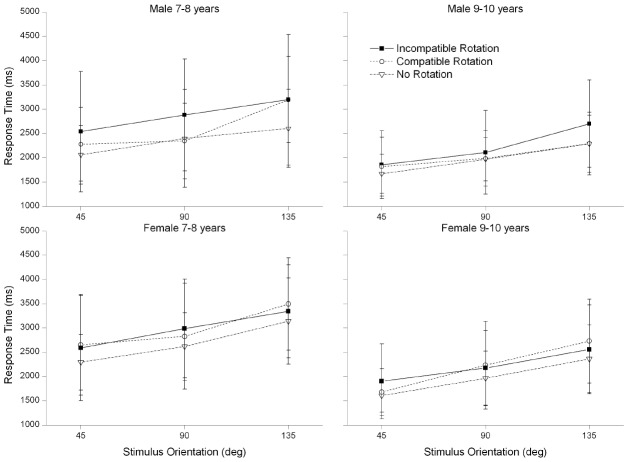
**Mean of the response times per age group and gender for compatible, incompatible, and no rotation trials**.

Main effects were found for “angular disparity,” *F*(2,148) = 53.15, *p* < 0.001, ${\text{η}}_{p}^{2}$ = 0.42, as well as for the factor “age group,” *F*(1,74) = 18.05, *p* < 0.01, ${\text{η}}_{p}^{2}$ = 0.20. Again, RT increased with increasing angle (repeated contrast analyses: all contrasts *p* < 0.001) and younger children had longer RT than older children (*M* = 2869 ms, SD = 113 vs. *M* = 2169 ms, SD = 120; see Figure 2). Additionally, significant interactions were found for the factors “age group” and “compatibility,” *F*(1,74) = 7.37, *p* < 0.01, ${\text{η}}_{p}^{2}$ = 0.09, “age group,” “compatibility,” and “gender,” *F*(1,74) = 8.35, *p* < 0.01, ${\text{η}}_{p}^{2}$ = 0.10 (see Figure 2), and for “compatibility” and “direction of manual rotation,” *F*(1,74) = 4.26, *p* < 0.05, ${\text{η}}_{p}^{2}$ = 0.05. For the latter interaction, *post hoc* analyses with *t*-tests revealed no significant differences between the mean RT during clockwise (*M* = 2491 ms, SD = 122) or counterclockwise (*M* = 2526 ms, SD = 119) manual rotations (*p* > 0.1).

To further investigate the interaction between “age group,” “compatibility,” and “gender,” separate analyses for each age group were calculated. In the younger age group the factor “compatibility” revealed a significant main effect, *F*(1,40) = 4.59, *p* < 0.05, ${\text{η}}_{p}^{2}$ = 0.10. In addition, a significant interaction was found between the factors “compatibility” and “gender,” *F*(1,40) = 5.89, *p* < 0.05, ${\text{η}}_{p}^{2}$ = 0.13. In the older age group, these effects were not found: “compatibility” (*p* = 0.09), interaction between “compatibility” and “gender” (*p* = 0.1). The compatibility effect can be accounted for by the boys in the younger age group (7- to 8-year-old boys: compatible rotation direction: *M* = 2613 ms, SD = 182; incompatible rotation direction: *M* = 2902 ms, SD = 190; 7- to 8-year-old girls: compatible rotation direction: *M* = 2989 ms, SD = 166; incompatible rotation direction: *M* = 2971 ms, SD = 172 and see Figure 2). To summarize, a significant effect of compatibility of rotation direction on the RT was found only for 7- to 8-year-old boys.

#### Hit Rates

To control for the influence of motor ability on compatibility effects, the M-ABC-2 overall score was added as a covariate. The ANCOVA yielded a main effect for the factor “age group,” *F*(1,74) = 6.13, *p* < 0.05, ${\text{η}}_{p}^{2}$ = 0.08. Older children had a higher HR than younger children (*M* = 95.8%, SD = 1.4 vs. *M* = 90.9%, SD = 1.3). Another main effect was found for the factor “angular disparity,” *F*(2,148) = 4.91, *p* < 0.01, ${\text{η}}_{p}^{2}$ = 0.06, with a higher HR for smaller disparities. Repeated contrast analyses revealed a significant difference between 45° and 90° (*p* < 0.05), but no significance for the difference between 90° and 135° (*p* > 0.05). For the factor “compatibility,” no significant effects (*p* > 0.05) or interactions were found (all *p* > 0.05). Finally, motor ability, as measured with the M-ABC-2, was significantly related to HR, *F*(1,74) = 5.0, *p* < 0.05, ${\text{η}}_{p}^{2}$ = 0.06. Thus, no significant effect of compatibility of rotation direction on HR was found.

### Effects of Motor Ability and Motor Priming on Subsequent Mental Rotation

Further analyses were performed to investigate whether a concurrent motor action (manually rotating a knob) primes the use of motor processes in a mental rotation task. Specifically, we asked whether motor processes are involved in mental rotation to a greater extent after performing a motor task in context with a mental rotation task. If this is the case, the RT and HR should differ between block 4 (mental rotation preceded by a motor task) and block 1 (mental rotation that was not preceded by a motor task).

To determine if motor ability should be used as a covariate to investigate this question, partial correlation analyses between the M-ABC-2 score and mental rotation performance (HR and RT) and “age in months” as a control variable were run in block 1 and 4. The Bonferroni-adapted partial correlations between M-ABC-2 score and mental rotation performance (HR and RT) were significant in the second block (block 4) of mental rotation (RT: *r* = –0.3, *p* < 0.01; HR: *r* = 0.3, *p* < 0.01) but not in the first block.

Two repeated measures ANCOVAs were subsequently run with the within-subjects factors “angular disparity” (0°, +45°, +90°, +135°, 180°, –135°, –90°, and –45°) and “priming” (with and without) and the between-subjects factors “gender” and “age group” for the dependent variables RT and HR; “M-ABC-2 overall score” was used as a covariate because of the correlation in block 4.

#### Response Time

The ANCOVA for the blocks of mental rotation without manual rotation with RT as dependent variable revealed main effects for “angular disparity,” *F*(7,504) = 4.89, *p* < 0.001, ${\text{η}}_{p}^{2}$ = 0.06, and “age group,” *F*(1,72) = 19.55, *p* < 0.001, ${\text{η}}_{p}^{2}$ = 0.21. RT increased with increasing angular disparity but repeated contrast analyses showed that the differences (*p* < 0.01) were significant only between 0° and 45° and between –45° and –90°. Older children had shorter RT than younger children (*M* = 1952 ms, SD = 95 vs. *M* = 2531 ms, SD = 90). No significant main effect for the factor “priming” was found (*p* > 0.05) indicating that no general learning effect occurred. A significant interaction was found between “angular disparity” and “gender,” *F*(7,504) = 2.3, *p* < 0.05, ${\text{η}}_{p}^{2}$ = 0.03. Separate analyses with *t*-tests showed significantly longer RT for girls only at 135° (girls: *M* = 2912 ms, SD = 966 vs. boys: *M* = 2391 ms, SD = 772). The family wise alpha error was below 5%. Another interaction was found between “priming” and “M-ABC-2 overall score,” *F*(1,72) = 4.01, *p* < 0.05, ${\text{η}}_{p}^{2}$ = 0.05. This interaction supports the correlation analysis reported at the beginning of the section: Children with more advanced motor skills show higher levels of performance in a mental rotation test only in the last block, i.e., after combined mental and manual rotation.

#### Hit Rates

In the ANCOVA with the dependent variable “HR,” the covariate “M-ABC-2 overall score” was significantly related to “HR,” *F*(1,78) = 5.96, *p* < 0.05, ${\text{η}}_{p}^{2}$ = 0.07. Significant main effects were also found for the factors “angular disparity,” *F*(7,546) = 5.73, *p* < 0.001, ${\text{η}}_{p}^{2}$ = 0.07, and “age group,” *F*(1,78) = 4.4, *p* < 0.05, ${\text{η}}_{p}^{2}$ = 0.05. HR decreased with increasing angular disparity but repeated contrast analyses showed that the only significant differences (*p* < 0.05) were between 0° and 45°, 45° and 90°, and –90° and –135°. Older children had higher HR than younger children (*M* = 93%, SD = 1.8 vs. *M* = 88%, SD = 1.6). No effect or interaction with the factor gender could be found (all *p* > 0.05).

There was also a significant interaction between “priming” and the covariate “M-ABC-2 overall score,” *F*(1,78) = 4.64, *p* < 0.05, ${\text{η}}_{p}^{2}$ = 0.06. *Post hoc* analysis of an interaction with a covariate is not possible. According to the correlation analysis reported at the beginning of the section, children with stronger motor abilities have shorter RT and higher HR. This holds true in the last block of mental rotation alone after two blocks with motor priming. There is no relationship found in the mental rotation block preceding the motor priming.

Another significant interaction was found between “angular disparity” and the covariate “M-ABC-2 overall score,” *F*(7,546) = 2.71, *p* < 0.01, ${\text{η}}_{p}^{2}$ = 0.03. Thus, the effect of motor priming on mental rotation performance depended on the overall score of the M-ABC-2. Children with advanced motor ability profited more from motor priming, i.e., performed better after combined mental and manual rotation than those with weaker motor ability.

## Discussion

The aim of the present experiment was to investigate effects of manual rotation on mental rotation in two different age groups and to test the impact of motor ability on these effects. A significant effect of compatibility of rotation direction on the RT in a mental rotation task was found only for 7- to 8-year-old boys. Rotating a knob in one direction interfered with the mental rotation of animal pictures in the opposite direction. Boys in the 7- to 8-year-old group were about 300 ms faster when mentally and manually rotating in the same direction compared to the incompatible condition. This effect could not be found for girls in the same age group or for 9- to 10-year-old children. An interaction between children’s motor abilities and the interference effect was not found. However, mean RTs and HRs in the mental rotation task were significantly influenced by children’s motor abilities after performing a manual rotation task (rotating a knob) in context with the mental rotation task.

### Mental Rotation

In line with previous literature, the findings of the present study include effects of both age and angular disparity on mental rotation performance (Kosslyn et al., 1990). Children in the younger age group made more errors and had longer RTs than children in the older age group. In both age groups, errors and RTs increased with increasing angular disparity. This result indicates that children did use mental rotation to solve the task (Shepard and Metzler, 1971). Although significant interactions were found between angular disparity and gender (RT) resp. manual rotation and gender (HRs), *post hoc* analyses did not reveal significant differences between boys and girls in any of the angular disparities and neither for the condition with, nor for the condition without manual rotation. This result is in contrast to the study of Jansen et al. (2013). A gender difference was only found when the effect of manual rotation compatibility was also assessed.

### Interference Between Motor Processes and Mental Rotation

Though the present study uses a slightly different paradigm, the results of Frick et al. (2009) were largely replicated. Compatible with our findings, Frick et al. (2009) found an effect of compatibility for younger children. Unlike Frick et al. (2009), the present results revealed an effect of gender. An age-dependent effect of compatibility supports the theory that the ability to dissociate visual mental activities and motor processes develops with age. The 7- to 8-year-old boys in our study showed a RT in the compatible condition that was around 300 ms shorter than in the incompatible condition. Moreover, the younger boys’ reaction time was around 300 ms shorter than that of the girls in the same age group. This gender difference was not expected and is, as far as we know, a new finding regarding dual task paradigms. As may be the case, boys take advantage of a strong relationship between motor and visual-mental processes as long as the task is not interfered by a concurrent motor task. This could possibly contribute to the explanation of the often found gender difference in mental rotation. However, with the data at hand, this point remains speculative. Please also note that no gender effects were found regarding HRs. Moreover, in contrast to our hypothesis, no interaction between motor ability and the compatibility effect could be found.

### Motor Ability, Mental Rotation, and Motor Priming

The influence of motor ability on the mean HRs is in line with previous literature (Jansen and Heil, 2010; Jansen et al., 2011b). According to Moreau (2012), the involvement of motor processes in non-motor processes, such as mental rotation, is due to prior extensive motor experience. Following their arguments, people with strong motor skills should be more likely to use motor processes while solving mental rotation tasks and profit from using these skills. In a different experiment, Wraga et al. (2003) showed that motor priming by performing a motor-related task has immediate consequences on a subsequent set of actions. The authors found that cortical areas in the brain that are involved in motor action were activated during mental rotation after motor priming. Hence, motor processes were used in computing the mental rotation of abstract objects. In contrast, these brain regions were not activated if the previous task included no motor priming.

A separate analysis of the RTs and HRs in the first block of mental rotation, where no motor priming in the form of manual rotation could trigger the involvement of motor processes, showed no influence of motor ability on mental rotation performance. In block 2 and 3 the HRs in the mental rotation task were significantly related to motor ability. Finally, in block 4, HRs and even RTs showed a relationship with children’s motor abilities. A general learning effect from block 1 to block 4 is unlikely because no main effect for the factor “priming” was found. A main effect would have indicated that all children improved their performance during the test. In contrast, the interaction between the factor priming and the covariate M-ABC-2 overall score shows that children’s mental rotation performance after the interference task was modulated by motor ability. Children with stronger motor ability profited more from simultaneous compatible manual and mental rotation. This suggests that the manual rotation of a knob in our experiment induced the use of motor processes to solve mental rotation tasks. Gender did not seem to play a crucial role in the analysis of priming effects. No gender effects were found for HRs. For RTs, a significant interaction between angular disparity and gender was found. However, separate analyses with *t*-tests showed significantly longer RT with girls for one angular disparity only.

Chu and Kita (2011) propose that the application of motor processes generally has a positive influence on mental processing of spatial transformations. Boys in the younger age group may have relied innately more on motor processes while solving the mental rotation task which proved beneficial and resulted in a mean RT that was around 300 ms shorter than the girls’ RT. But if this reliance on learned motor processes was disrupted by a concurrent motor process such as rotating a knob in the opposite direction, boys had to rely more on visual processes. This might result in a mean RT of the same length as the girls’. Whether the girls in the 7-to 8-year-old age group relied on visual processes while solving mental rotation tasks cannot be derived from these data, since the concurrent motor task increased girls’ RT and it was not influenced by direction of manual rotation.

We may only speculate about why gender differences were found for the effect of compatibility in the younger age group. One reason might be that the boys in this age group had a better action-perception coupling (Piaget, 1952; Mounoud et al., 2007). “Action-perception coupling” refers to the observation made by Mounoud et al. (2007) that the perception of an action pantomime can facilitate the subsequent recognition of a corresponding tool. Given boys’ general preference for toys which tend to encourage manipulation, construction, and active exploration (Cherney and London, 2006) and thus foster spatial abilities (Robert and Héroux, 2004), 7-to 8-year-old boys may be more sensitive to effects of compatibility. For the children in the older age group, faster RTs, higher HRs and no effects of compatibility were found. This supports the idea that as children grow older, there is a developmental shift that allows for better decoupling of visual mental representations and manipulations on the one hand and motor processes on the other.

### Limitations

Some limitations of the study should be noted. In the paradigm used, an arrow appeared on the screen indicating the direction the knob should be rotated in. As soon as the knob was rotated in the correct direction the arrow disappeared and the stimuli for the mental rotation task appeared on the screen. Children were told to constantly rotate the knob while solving the mental rotation task. When cognitive load increased while solving the mental rotation task, many children slowed their speed of manual rotation or even stopped. Although children were reminded of the instructions when this was observed, they soon returned to this behavior. In further studies it may prove effective to couple the knob with a velocity detection system so that a possible slowing of the rotation can be measured. Nevertheless, a compatibility effect was observed in the present study and the use of motor processes in solving a mental rotation could be induced.

The possibility that some children, in contrast to the instructions, might have rotated the left (upright) stimulus in order to align it with the right (rotated) stimulus cannot be ruled out. Another point that has to be considered is that the presentation of the arrow might have stimulated a predominantly visual strategy to solve the mental rotation task thus reducing interference effect due to motor processes. This point can also not be ruled out completely with our data. The finding that mental rotation performance in the block subsequent to the manual mental rotation task is clearly influenced by motor ability, however, shows that beneficial motor processes have been induced in children with stronger motor skills. Further research with the arrow as a primer prior to mental rotation without manual rotation might resolve this issue. Furthermore, it may be possible that it was primarily girls who stopped rotating the knob.

## Conclusion

The collective results of this study suggest that 7- to 8-year-old boys rely more on motor processes in solving mental transformation tasks compared to girls of the same age. In older children, this difference may be eliminated due to more advanced cognitive skills, but this theory should be investigated in further studies. Children with strong motor abilities are more likely to use beneficial motor processes in mental rotation tasks after performing a motor task in context with a mental rotation task. These results confirm an overlap between motor and cognitive processes, especially for young children, and underline the importance of multifaceted motor experience.

### Conflict of Interest Statement

The authors declare that the research was conducted in the absence of any commercial or financial relationships that could be construed as a potential conflict of interest.
